# Behavioral problems in children with epilepsy in rural Kenya

**DOI:** 10.1016/j.yebeh.2011.10.017

**Published:** 2012-01

**Authors:** Symon M. Kariuki, Amina Abubakar, Penny A. Holding, Victor Mung'ala-Odera, Eddie Chengo, Michael Kihara, Brian G. Neville, Charles R.J.C. Newton

**Affiliations:** a Center for Geographic Medicine Research—Coast, Kenya Medical Research Institute, Kilifi, Kenya; b Tilburg University, Tilburg, The Netherlands; c Utrecht University, Utrecht, The Netherlands; d International Centre for Behavioral Studies, Kenya; e Neuroscience Unit, University College London, Institute of Child Health, London, UK; f Clinical Research Unit, London School of Hygiene and Tropical Medicine, London, UK; g Department of Psychiatry, University of Oxford, Oxford, UK

**Keywords:** Behavioral problems, Children, Cognitive impairment, Epilepsy, Kenya

## Abstract

The aims of this study were to record behavioral problems in children with epilepsy (CWE), compare the prevalence with that reported among healthy children without epilepsy, and investigate the risk factors. A child behavioral questionnaire for parents comprising 15 items was administered to the main caregiver of 108 CWE and 108 controls matched for age in Kilifi, Kenya. CWE had a higher mean score for reported behavioral problems than controls (6.9 vs 4.9, *t* = 4.7, *P* < 0.001). CWE with active epilepsy also recorded more behavioral problems than those with inactive epilepsy (8.2 vs 6.2, *t* = − 2.9, *P* = 0.005). A significantly greater proportion of CWE (49% vs 26% of controls) were reported to have behavioral problems. Active epilepsy, cognitive impairment, and focal seizures were the most significant independent covariates of behavioral problems. Behavioral problems in African CWE are common and need to be taken into consideration in planning comprehensive clinical services in this region.

## Highlights

► Behavioral problems in children with epilepsy were studied. ► Behavioral problems were reported in 49% of children with epilepsy and in 26% of the controls. ► Active epilepsy type, cognitive impairment, and focal seizures predicted behavioral problems. ► Behavioral problems are common in children with epilepsy and need to be considered in planning clinical services.

## 1 Introduction

Psychiatric conditions such as attention-deficit/hyperactivity disorder (ADHD), autistic spectrum disorders (ASD), and affective, aggressive, and social disorders occur in children with epilepsy (CWE) and have a major influence on the quality of life of these children [1–4]. CWE exhibit substantially more behavioral problems, particularly those relating to social activity, attention, and problem solving [5,6], than do children with chronic non-neurological conditions such as diabetes, asthma, heart diseases, and rheumatoid arthritis.

A high frequency of behavioral problems in CWE was reported in earlier studies from developed countries [7], and recent studies from these countries confirm that at least a third of CWE have a psychiatric diagnosis [8–10]. Although most of these studies are population based, they may not provide a universally/geographically representative sample as none of these studies come from resource-poor settings, including sub-Saharan Africa, despite the high burden of epilepsy in these areas [11]. Differences in symptomatic etiologies, treatment practices, and survival may modify cognitive and behavioral outcomes in the CWE in these regions. Infectious encephalopathies and head trauma are more common [12–14] and outcome may be influenced by different therapeutic regimes. The antiepileptic drugs (AEDs) used in resource-poor settings, such as phenobarbital and carbamazepine, have, in some studies, been associated with an increased prevalence of behavioral problems in children [15,16]. Additional data are therefore required to provide a more informed picture of the size of the problem across different health care contexts.

Much remains to be understood concerning the etiology of behavioral problems in epilepsy. The literature is inconsistent, but suggests both biomedical and psychosocial risk factors for behavioral problems in CWE [5,17]. Potential contributors to variability in outcome have included, type, severity, and duration of epilepsy [18], parenting processes and family dynamics [19], and individual characteristics such as early temperament and cognitive performance levels [20,21]. Associated risk factors that explain variability in outcome in resource-poor countries are yet to be explored.

We investigated behavioral problems in older CWE, in whom we have reported a high prevalence (41 per 1000) and incidence (182 per 100,000/year) of epilepsy in children aged between 6 and 9 years in Kilifi, Kenya [22]. To understand the long-term burden of behavioral problems on the children and their families, we studied children above 6 years of age, as they survived early childhood, a period with high mortality in sub-Saharan Africa [23]. The aims of this study were to record behavioral problems in CWE, compare the prevalence of behavioral problems with that reported among healthy children without epilepsy, and investigate factors associated with behavioral problems in CWE. These data are envisaged to provide a basis for planning comprehensive clinical interventions for CWE.

## 2 Materials and methods

### 2.1 Study settings

The study was conducted in Kilifi District, a rural area on the coast of Kenya. The Mijikenda community formed the majority of the study population. Kilifi District is among the poorest in Kenya, with a low literacy level and poor access to sanitation facilities [24]. Most of the people are subsistence farmers or fishermen. Malaria, pneumonia, and bacteremia are the major causes of pediatric admissions to Kilifi District Hospital (KDH) [13,25]. The adjusted prevalence of active convulsive epilepsy in all ages during the time of the study was 4.5 per 1000 (95% CI: 4.1–4.9) [24]. During the same period, the epilepsy treatment gap, defined as the proportion of people across all ages with epilepsy who require treatment and do not receive it, was 70% [24]. This may be an underestimate because people conceal their epilepsy [26], as people from this area still believe epilepsy is caused by supernatural spirits [27].

Epilepsy services are provided both in KDH and at the epilepsy clinic at the Kenya Medical Research Institute (KEMRI)/Wellcome Trust Research Programme. In particular, AEDs such as phenobarbital, phenytoin, carbamazepine, and sodium valproate, as well as counseling services, are provided free. However, a substantial number of people with epilepsy seek medical care from traditional healers who attribute epilepsy to supernatural spirits and are easily accessible [27]. The traditional healers treat epilepsy either by forcefully driving spirits out using herbs or foul-smelling concoctions or by enticing them out though offering of sacrifices [27].

### 2.2 Study population

Children born between 1 June 1991 and 31 December 1995, and thus aged 6–9 years, were identified from a survey conducted between June 2001 and April 2002 on neurological impairment and disability [28]. This survey identified epilepsy as the most common impairment, followed by cognition, hearing, motor, and visual impairments. The behavioral problems for children with epilepsy from this survey are reported in this analysis. We selected children 6 years and older because of the difficulty of differentiating febrile seizures and unprovoked seizures in younger children and because these older children, having survived the earlier years, provide a picture of the longer-term effects of epilepsy [23].

The study population for this analysis consisted of 110 CWE (73 with inactive epilepsy and 35 with active epilepsy) and 110 children identified from the same population database and matched on sex and age who had neither epilepsy nor any previous admission with encephalopathy.

### 2.3 Assessments

#### 2.3.1 Epilepsy

The diagnosis of epilepsy was based on a clinical history of two or more unprovoked seizures as confirmed by a clinician. A 30-minute EEG was obtained to classify the seizures. Active epilepsy was defined according to the criteria for administration of AEDs in Kenya [29]: more than one unprovoked convulsion with at least one in the last 12 months. Inactive epilepsy was defined as a lifetime history of more than one unprovoked seizure, with none in the preceding year, and this included even seizure-free individuals taking AEDs. Lifetime epilepsy included both active and inactive epilepsy.

#### 2.3.2 Behavior

The Child Behavior Questionnaire for Parents (CBQFP) was used to ascertain the existence and level of behavior problems. The questionnaire consists of 15 items each assessing a different domain of behavior, including aggression, socialization, reaction to change, worries and fears, and habitual behaviors. The questionnaire was administered to the main caregiver of the child; mothers were the most common respondents (181/216, 84%). The main caregivers were questioned in a conversational manner, and the interviewer rated responses according to the extent and regularity of the behavior described. Scores were summed, with a higher overall score denoting a greater level of behavior problems [30]. The tool had been used previously to investigate the association between severe malaria infection and neurobehavioral outcome [30,31]. In this study, the behavioral tool demonstrated relatively good reliability (Cronbach's α = 0.72), and average inter-item covariance was 0.04 for CWE. The cutoff for behavioral problems was defined as scores in the top third of the normative group (*n* = 1107) selected from the community who did not have epilepsy or a history of encephalopathy, following guidelines provided by Richman et al. [32]. The top third of CBQFP questionnaire scores of the normative group was used in the present study, as it is similar in context and format to the behavioral questionnaire developed by Richman et al. and this criterion was used to identify preschool- and school-aged children requiring referral to child mental health services [33].

#### 2.3.3 Covariates

##### 2.3.3.1 Cognition

An assessment of cognitive development was performed on all cases and controls using an assessment battery of seven tests measuring verbal and nonverbal skills developed for children in the area. The previously described battery comprised: Category Fluency (verbal processing,) Information, and Picture Vocabulary (verbal knowledge), Panga Muntu- Construct-a-Man (intellectual maturity), Matching Familiar Figures (processing speed), and Construction (visuospatial organization) [28,34]. Children's cognitive development was described as either impaired or unimpaired. Impaired cognitive development was defined by a score at or below 2SD of the normative group mean in two or more tests.

##### 2.3.3.2 Other background covariates

A (trained) field worker and a clinician collected information about the possible covariates of behavioral scores using a sociodemographic and neurological assessment questionnaire, respectively. The factors included in the analysis were grouped into family characteristics, epilepsy, and medical history or status. Family characteristics studied were child's age (as a continuous variable), sex (females coded as 1 and males as 0), place of delivery (home coded as 1 and hospital as 0), and attendance at school (non-attendance coded as 1 and attendance as 0), and parent's marital status (single coded as 1 and non-single as 0), income and age (as a continuous variable); and number of children in the family (as a continuous variable). The epilepsy factors investigated included epilepsy type (active coded as 1 and inactive as 0), seizure frequency (as a continuous variable), focal seizures (focal coded as 1 and generalized as 0), use of AEDs (use coded as 1 and non-use as 0), interictal epileptiform discharges (present coded as 1 and absent coded as 0), and age at onset of seizures (as a continuous variable). Under medical history or status, we investigated nutritional status (body mass index [BMI] < 18.5 coded as 1 and BMI ≥ 18.5 coded as 0), cognitive impairment (impairment coded as 1 and lack of impairment coded as 0), neurological status (presence of neurological deficits coded as 1 and absence of neurological deficits coded as 0), status of immunization (incomplete coded as 1 and complete coded as 0), history of birth insults (positive coded as 1 and negative coded as 0), history of neonatal jaundice (positive coded as 1 and negative coded as 0), and attainment of developmental milestones (abnormal coded as 1 and normal coded as 0). Attainment of developmental milestones was classified as either normal or abnormal from the history taken by a (trained) clinician (blind to the cognitive performance scores) from all CWE. Abnormal development characterized those children who had had delay in sitting, standing, or walking or motor, language, and cognitive milestones and who could not learn to do the things healthy children of a similar age did. All the factors considered here have been previously associated with behavioral problems in the literature or have been anecdotal evidence of association with behavioral problems in our clinical practice.

### 2.4 Statistical analysis

Data were analyzed using STATA (Version 11; Stata Corp, TX, USA). We used Student's *t* test to compare scores on the CBQFP between the CWE and the control group and, in a subanalysis, between active epilepsy and inactive epilepsy. We used the Mann–Whitney two-sample statistic to compare scores for each behavioral item (scores were not normally distributed) between the cases and controls. Pearson's *χ*^2^ test was used to compare cognitive impairment and other categorical variables between the CWE and the control group and, in a subanalysis, between active epilepsy and inactive epilepsy. Linear regression was used to identify significant covariates of total behavioral scores (as a continuous variable) within the CWE, whereas logistic regression was used to determine the factors associated with the probability of developing behavioral problems in CWE (behavioral problems defined as the proportion of children with behavioral scores in the top third of the normative sample scores as described above). Those background factors that had a univariate *P* value ≤ 0.25 were entered into a multivariate linear or logistic regression to identify independent factors through a backward elimination process, in which the largest *P* value was removed until all the remaining variables made significant partial contributions to the final model according to the usual *t* test or *F* test. There was significant collinearity (strong correlation) between cognitive impairment and history of abnormal developmental milestones (*r* = 0.51, *P* < 0.001), and the latter was therefore not included in the multivariate regression model. Analysis of variance was used to measure the difference in distribution of behavioral scores among the eight seizure types documented in CWE.

Permission to conduct this study was granted by the KEMRI National Research and Ethical Committee, and informed consent was elicited from the parent or guardian of the participating child.

## 3 Results

### 3.1 General description

The CBQFP was incomplete for two CWE from the study sample and thus we report results for 108 CWE and 108 controls. Fifty percent of the pairs were boys. There were no significant differences between CWE and controls on background characteristics measured, except for the health indicators (number of previous hospitalization, previous hospitalization with seizures, and history of birth difficulties) (Table 1). EEGs were obtained for 78 CWE, and interictal epileptiform discharges were demonstrated in 14 (17.9%) of these children. The proportion of interictal epileptiform discharges did not statistically differ between children with active epilepsy and those with inactive epilepsy (*χ*^2^[1] = 0.031, *P* = 0.955).

Only four children with active epilepsy were taking AEDs, and none with inactive epilepsy were taking AEDs (*χ*^2^[1] = 8.66, *P* = 0.003). Significantly fewer children with active epilepsy than with inactive epilepsy were regularly attending school (13/35 [37.1%] vs 43/73 [58.9%], *χ*^2^[1] = 4.49, *P* = 0.034). A greater number of children with active epilepsy had generalized absence seizures compared with those with inactive epilepsy (4/35 [11.4%] vs 0/73 [0%], *χ*^2^[1] = 8.66, *P* = 0.003). Children with active epilepsy and those with inactive epilepsy did not statistically differ on other clinical and sociodemographic characteristics.

### 3.2 Behavioral scores and/or problems and cognition in children with epilepsy

Behavioral problems were found in 52 (49%) CWE and 28 (26%) controls (*χ*^2^[1] = 11.44, *P* < 0.001). CWE had significantly higher mean total behavioral scores than controls (6.9 vs 4.9, *t* = 4.7, *P* < 0.001). There was no difference in behavioral scores among the eight different seizure types (*F* = 0.48, *P* = 0.946). Despite the small numbers, the relationship between AEDs and behavior scores was investigated for trends. There was only a 1-point difference in total behavioral scores between those children on AEDs and those not on medication (*t* = − 0.37, *P* = 0.714). However, the three CWE on phenorbarbital had worse behavioral scores than the one on carbamazepine (8.7 vs 4.0), but the numbers were too small for any meaningful statistical comparison. There was no difference between girls and boys with epilepsy on mean total behavioral scores (6.8 vs 6.9, *t* = − 0.14, *P* = 0.886). Total behavioral scores were similar in both children with interictal epileptiform discharges on the EEG and those without (6.6 vs 7.0, t = − 0.39, *P* = 0.697). The proportion of children with cognitive impairment was 30.6% in CWE and 5.6% in the controls (*χ*^2^[1] = 22.81, *P* < 0.001).

Mean total behavioral scores were worse in children with active epilepsy than in those with inactive epilepsy (8.2 vs 6.2, t = − 2.87, *P* = 0.005). Cognitive impairment was more common in children with active epilepsy than in those with inactive epilepsy, although with a borderline statistical significance (15/35 [42.9%] vs 18/73 [24.7%], *χ*^2^(1) = 3.69, *P* = 0.055).

Children with epilepsy had statistically higher behavioral scores than controls on 8 of the 15 behavioral items investigated in this analysis. Post hoc analysis at an item-by-item level suggests that this difference is due to a combination of the following features of behavior: concentration span (*z* = 3.7, *P* < 0.001), social relationships (*z* = 3.9, *P* < 0.001), self-care (*z* = 3.3, *P* = 0.001), temper or tantrums (*z* = 3.0, *P* = 0.003), habits (*z* = 2.4, *P* = 0.013), dependence on caretakers (*z* = 2.1, *P* = 0.038), empathy (*z* = 2.2, *P* = 0.036), and fears (*z* = 1.9, *P* = 0.052).

### 3.3 Covariates of behavioral problems and/or scores in children with epilepsy

Univariate linear regression identified cognitive impairment (β coefficient = 3.07, *P* < 0.001), frequency of seizures (β = 0.27, *P* = 0.010), active epilepsy (β = 2.02, *P* = 0.005), neurological deficits on clinical examination (β = 1.75, *P* = 0.016), and history of abnormal developmental milestones (β = 2.71, *P* = 0.001) as the factors positively associated with behavioral scores (Table 2). In the multivariate model, cognitive impairment (β = 2.95, R^2^ = 0.32, *P* = 0.012) and active epilepsy (β = 1.95, R^2^ = 0.32, *P* = 0.030) remained as single factors associated with total behavioral scores.

The univariate results for the predictors of development of behavioral problems are listed in Table 3. Cognitive impairment (odds ratio [OR] = 4.48, 95% CI: 1.82–10.99, *P* = 0.001) and history of abnormal developmental milestones (OR = 3.26, 95% CI: 1.24–8.53, *P* = 0.016) were associated with development of severe behavioral problems in CWE in the univariate logistic regression analysis. However, in the multivariate logistic regression analysis, focal seizures (OR = 13.92, 95% CI: 1.57–123.87, *P* = 0.018) and active epilepsy (OR = 7.86, 95% CI: 1.23–50.06, *P* = 0.029) appeared as the independent predictors of behavioral problems in CWE.

## 4 Discussion

This is the first epidemiological study known to have been conducted in sub-Saharan Africa to systematically investigate behavioral problems in school-aged CWE. Similar to researchers in other settings, we found that behavioral problems were more common in CWE than in children without epilepsy, most particularly in children with active epilepsy. The frequency of behavioral problems suggests that the problem may be worse in resource-poor settings than in temperate geographical regions, where estimates are at around 30% [18,35]. This difference is likely to be caused by the high level of untreated epilepsy [24] and the high incidence of symptomatic causes of epilepsy [12,14], which may modify the encephalopathies associated with behavioral problems. Our results are analogous to those of studies from settings with similar health and social factors such as Thailand and India, where reported behavioral problems occurred in 57 and 54% of CWE, respectively [36,37]. Of the types of behavioral problems that were particularly common in the CWE studied, concentration span and social relationships have also been found in other studies to be commonly associated with epilepsy [38].

### 4.1 Cognitive impairment and behavioral problems

Although we recorded fewer CWE with cognitive impairment than behavioral problems, in common with previous studies, cognitive impairment was still more prevalent in CWE than in the control children [39]. This suggests that the diagnosis and management of behavioral problems should be a primary consideration in clinical services provided to those with epilepsy, and should be integrated with appropriate educational support. The significant association between cognitive impairment and behavioral scores demonstrated in the multivariate analysis further suggests that educational and behavioral support is required by many CWE [32]. Furthermore, the association observed between cognition and behavior may be explained by the influence of seizure activity (either convulsive or nonconvulsive) on the development of cognitive functions in children during the period of brain plasticity [40]. The role of seizure activity in cognitive function is supported by the finding from our present study that active epilepsy was independently associated with behavioral problems in CWE. Some have speculated that the loss of cognitive skills may manifest as behavioral problems because cognitive ability may be important in learning to program adaptive behaviors (age-appropriate behaviors necessary for children to live independently in new situations) [20], and this could also apply to CWE. Additionally, the nature of the underlying brain disease that gives rise to epilepsy may also be a cause of both cognitive impairment and behavioral problems in CWE [40]. The independent association in our multilevel model between active epilepsy and behavioral problems and cognitive impairment and total behavioral scores further supports the close association between these three factors; seizure activity, cognition, and behavioral development.

### 4.2 Effect of antiepileptic drugs on behavioral problems

Because of the large treatment gap for epilepsy in this area [24], only four CWE were taking AEDs. The sample size is too small to analyze differences in behavior between those taking AEDs and those not taking AEDs. Evidence, mainly from resource-rich countries, has pointed to phenobarbital as causing features of ADHD, but more comprehensive studies in low-resource settings have failed to confirm this in Kenya [41], in India [42], and particularly in a randomized controlled trial between phenobarbital and carbamazepine in Bangladesh [43]. However, clinical and educational interventions aimed at reducing the large treatment gap for epilepsy in this area are still needed [24], as this may result in control of seizures, and the associated cognitive and behavioral problems, in CWE. Additional studies on behavioral outcomes of the use of various AEDs in Africa are probably justified.

### 4.3 The significance of seizure activity

Both behavioral problems and cognitive impairment were more common in children with active epilepsy than in those with inactive epilepsy. This is further supported by the association between active epilepsy and behavioral problems in the multivariate analysis. It was therefore unsurprising that children with active epilepsy were also less likely to attend school regularly. The irregular attendance at school may also be due to overprotective parents, who will not allow their ill children to be alone at school. As discussed above, although both behavioral problems and cognitive impairment may have a common etiology associated with repetitive seizure activity [40], there are inconsistencies in the literature investigating the relationship between seizure activity and developmental outcome. While some studies support the direct influence of seizure activity [20], others do not [1,44]. The discrepancy among studies can be explained by possible differences in study designs, failure of patients to recall seizure frequency, and differences in epilepsy syndromes or seizure types studied. Use of standard seizure classification criteria across all studies may help resolve some of the documented discrepancies.

Although we did not find any difference in total behavior scores among the different seizure types, we found an association between focal seizures and the development of severe behavioral problems. This is in agreement with some studies that have identified seizure type as specifically affecting aggression scores, but not general social competence or total behavior scores [45]. As in our study, Millichap found more general behavioral problems in children who had experienced focal seizures than in those who had generalized seizures [46]. We may have underestimated the effect of focal seizures on behavioral problems, as many focal seizures that generalize are classified as generalized seizures in Africa [47]. Future studies could focus on clarifying further the specific nature of the problems encountered and the possible etiology of these outcomes.

### 4.4 Family characteristics and behavioral problems

Although Pianta et al. found parenting and family characteristics to be important predictors of behavioral problems in epilepsy [48], our findings show that family factors alone cannot be used to explain behavioral problems in CWE. Given the methodology we used to document behavior problems, parental report, family perceptions, and family processes may have contributed to the differential rates reported both between CWE and the reference sample and between those with active and those with inactive epilepsy. The greater prevalence of behavior problems as recorded by parental report may also be in part attributable to the concerns of parents rather than specific behavioral impairments. The possible influence of parental concerns on the rate of problems reported, as well as on decisions on whether to send children to school or not, suggests two future directions for research and intervention. First, the validation through multiple respondents and direct observation of parental report may clarify the objectivity or otherwise of the methodology. There is also the suggestion that family education may be used to supplement treatment in comprehensive management of CWE, leading to a reduction in both behavioral problems and the consequences of a lack of access to education for CWE [36,39].

### 4.5 Limitations

In any survey, respondents may suffer recall bias, and others may exaggerate the extent of behavioral problems; a multireporter approach to gathering information may reduce these effects. In addition, a larger sample size would have yielded more power to detect the differences in behavioral problems in subgroup analysis. Because of the large treatment gap in our study area and the resulting small number of children taking AEDs, we were unable to evaluate the impact of AED regimes. Future prospective studies in this area need to investigate the extent to which epilepsy contributes to the development of behavioral problems and cognitive impairment in CWE in the presence of other potential risk factors.

## 5 Conclusions

Our results provide an important estimate of the burden of behavioral problems in CWE in sub-Saharan Africa. The children we studied came from a community sample and provide a more representative sample than those attending epilepsy clinics, who tend to have more severe disease. The association of seizure frequency and cognitive impairment with behavioral problems in CWE suggests a need for a comprehensive clinical and educational support program for CWE and their families in the community.

## Conflict of interest statement

We confirm that there are no conflicts of interest to disclose.

## Figures and Tables

**Table 1:** Characteristics of study participants.

Characteristic	Cases	Controls	Statistical difference
(*N* = 108)	(*N* = 108)
Age, months	7.4 [1.14]^a^	7.4 [1.14]	*t* = − 0.06, *P* = 0.95
Males	54 (50.0%)	54 (50.0%)	*χ*^2^ = 0.00, *P* = 1.0
Irregular attendance at school	52 (48.1%)	47 (54.5%)	*χ*^2^ = 0.30, *P* = 0.59
Number of children in family	5.8 [2.5]	5.8 [2.4]	*t* = − 0.07, *P* = 0.94
Single maternal marital status	21/104 (20.2%)	28/102 (27.5%)	*χ*^2^ = 1.50, *P* = 0.23
Mother's lack of income-generating activity	67/104 (44.4%)	61/102 (59.8%)	*χ*^2^ = 0.45, *P* = 0.49
Father's lack of income-generating activity	31/92 (33.7%)	22/83 (26.5%)	*χ*^2^ = 1.07, *P* = 0.30
Mother's age, years	32.1 [5.9]	32.2 [6.6]	*t* = − 0.21, *P* = 0.84
Malnourished (BMI < 18.5)	38/107 (35.5%)	31/107 (28.9%)	χ ^2^ = 1.05, *P* = 0.31
Cognitive impairment	33 (30.6%)	6 (5.6%)	*χ*^2^ = 22.80, *P* < 0.01
History of abnormal developmental milestones	27/91 (29.7%)	17/92 (18.5%)	*χ*^2^ = 3.14, *P* = 0.08
History of birth difficulties	38 (35.2%)	6 (5.6%)	*χ*^2^ = 29.22, *P* < 0.001
History of neonatal jaundice	13 (12.0%)	7 (6.5%)	*χ*^2^ = 1.98, *P* = 0.16
Incomplete immunization	5 (4.6%)	7 (6.5%)	*χ*^2^ = 0.35, *P* = 0.55
Previous hospitalization	78 (72.2%)	38 (35.1%)	*χ*^2^ = 28.32, *P* < 0.01
Previous hospitalization with seizures	62 (57.4%)	0	*χ*^2^ = 84.18, *P* < 0.01

a Values expressed as either mean [SD] or number (%).

**Table 2:** Results of univariate linear regression analysis of background covariates of total behavioral scores in children with epilepsy.

Covariate	*R*^2^, *F* test	β coefficient, *t* test
Family or sociodemographic factors		
Mother's age	*R*^2^ < 0.01, *P* = 0.87	β = 0.01, *P* = 0.87
Child's sex (male)	*R*^2^ < 0.01, *P* = 0.89	β = 0.10, *P* = 0.89
Number of children born to family	*R*^2^ < 0.01, *P* = 0.81	β = − 0.03, *P* = 0.81
Maternal level of education	*R*^2^ = 0.04, *P* = 0.14	β = 0.33, *P* = 0.14
Delivery at home	*R*^2^ < 0.01, *P* = 0.78	β = − 0.22, *P* = 0.78
Father's lack of income-generating activity	*R*^2^ = 0.01, *P* = 0.27	β = 0.86, *P* = 0.27
Single maternal marital status	*R*^2^ = 0.01, *P* = 0.42	β = − 0.25 *P* = 0.42
Irregular attendance of school	*R*^2^ = 0.02, *P* = 0.15	β = − 0.97, *P* = 0.16
Mother's lack of income-generating activity	*R*^2^ = 0.02, *P* = 0.14	β = 1.05, *P* = 0.14
Child's age	*R*^2^ < 0.01, *P* = 0.98	β = 0.01, *P* = 0.98
Epilepsy factors		
Seizure frequency	*R*^2^ = 0.07, *P* < 0.01	β = 0.27, *P* = 0.01
Focal seizures	*R*^2^ < 0.01, *P* = 0.69	β = 0.41, *P* = 0.69
Active epilepsy	*R*^2^ = 0.07, *P* < 0.01	β = 2.02, *P* < 0.01
Antiepileptic drug use	*R*^2^ < 0.01, *P* = 0.72	β = 0.66, *P* = 0.72
Interictal epileptiform discharges on EEG	*R*^2^ < 0.01, *P* = 0.74	β = − 0.34, *P* = 0.70
Medical history or status		
Age at onset of seizures	*R*^2^ = 0.02, *P* = 0.23	β = − 0.02, *P* = 0.23
Malnutrition (BMI < 18.5)	*R*^2^ < 0.01, *P* = 0.58	β = − 0.05, *P* = 0.58
Neurological deficits on clinical examination	*R*^2^ = 0.05, *P* = 0.02	β = 1.75, *P* = 0.02
Cognitive impairment	*R*^2^ = 0.16, *P* < 0.01	β = 3.04, *P* < 0.01
Abnormal developmental milestones	*R*^2^ = 0.12, *P* < 0.01	β = 2.7, *P* < 0.01
Incomplete immunization	*R*^2^ < 0.01, *P* = 0.55	β = 0.99, *P* = 0.55
History of difficult birth	*R*^2^ < 0.01, *P* = 0.72	β = 0.26, *P* = 0.72
History of neonatal jaundice	*R*^2^ = 0.09, *P* < 0.01	β = 3.31, *P* < 0.01

*Note.* Linear regression was used to measure the prediction of high behavioral scores in children with epilepsy by each covariate. *R*^2^ and the corresponding *P* value are a measure of the good-fitness of the model. β coefficient and the corresponding *P* value are a measure of the direction and strength of prediction of high behavioral scores by each covariate in children with epilepsy.

**Table 3:** Results of univariate logistic regression analysis of background covariates of behavioral problems in children with epilepsy.

Covariate	Odds ratio (95% CI)	*P* value
Family or sociodemographic factors		
Mother's age	1.00 (0.93–1.06)	0.90
Child's sex (male)	1.25 (0.59–2.67)	0.56
Number of children born to the family	0.99 (0.85–1.15)	0.90
Maternal level of education	1.10 (0.88–1.38)	0.42
Delivery at home	1.11 (0.46–2.69)	0.82
Father's lack of income-generating activity	1.99 (0.83–4.82)	0.13
Single maternal marital status	0.63 (0.24–1.17)	0.36
Irregular attendance at school	0.93 (0.63–1.37)	0.71
Mother's lack of income-generating activity	1.80 (0.80–4.08)	0.16
Child's age	0.82 (0.59–1.15)	0.26
Epilepsy factors		
Seizure frequency	1.08 (0.96–1.22)	0.20
Focal seizures	2.13 (0.67–6.85)	0.20
Active epilepsy	1.71 (0.76–3.85)	0.20
Antiepileptic drug use	1.08 (0.15–7.95)	0.94
Interictal epileptiform discharges on EEG	0.50 (0.15–1.68)	0.26
Medical history or status		
Age at onset of seizures	0.99 (0.97–1.00)	0.14
Malnutrition (BMI < 18.5)	1.36 (0.62–3.01)	0.45
Neurological deficits on clinical examination	1.32 (0.59–2.99)	0.50
Cognitive impairment	4.48 (1.82–10.99)	< 0.01
Abnormal developmental milestones	3.26 (1.24–8.53)	0.02
Incomplete immunization	1.65 (0.27–10.3)	0.59
History of a difficult birth	1.55 (0.70–3.44)	0.28
History of neonatal jaundice	2.72 (0.78–9.45)	0.12

*Note.* Logistic regression was used to measure the prediction of behavioral problems in children with epilepsy by each covariate. Behavioral problems were defined as the proportion of children with epilepsy with total behavioral scores in the top third of the normative group's total behavioral scores.
